# Hydrogen Sulfide Interacting with Cannabinoid 2 Receptors during Sciatic Nerve Injury-Induced Neuropathic Pain

**DOI:** 10.3390/antiox12061179

**Published:** 2023-05-30

**Authors:** Xue Bai, Gerard Batallé, Ignacio Martínez-Martel, Olga Pol

**Affiliations:** 1 Grup de Neurofarmacologia Molecular, Institut d’Investigació Biomèdica Sant Pau (IIB Sant Pau), 08041 Barcelona, Spain; 2 Grup de Neurofarmacologia Molecular, Institut de Neurociències, Universitat Autònoma de Barcelona, 08193 Barcelona, Spain

**Keywords:** analgesia, anxiety, BDNF, cannabinoids, cannabinoid 2 receptors, depression, hydrogen sulfide, neuropathic pain, oxidative stress

## Abstract

Hydrogen sulfide (H_2_S) donors make opioids more effective in inhibiting nociception during inflammatory and neuropathic pain. We examined whether the analgesic, anxiolytic and/or antidepressant actions of the cannabinoid 2 receptor (CB2R) agonist, JWH-133, might be improved by pretreatment with H_2_S donors, DADS and GYY4137 in mice with sciatic nerve injury-provoked neuropathy (CCI). The reversion of the antinociceptive effects of these treatments with the CB2R antagonist, AM630, and the regulatory actions of H_2_S in the phosphorylation of NF-κB inhibitor alpha (IKBα) and in the brain-derived neurotrophic factor (BDNF), CB2R, Nrf2 and heme oxygenase 1 (HO-1) levels in prefrontal cortex (PFC), ventral hippocampus (vHIP) and periaqueductal gray matter (PAG), were examined. Data showed that the analgesic effects of JWH-133, systemically and locally administered, were improved by the DADS or GYY4137 pretreatment. The co-treatment of GYY4137 with JWH-133 also stopped anxiodepressive-like activities that concur with neuropathy. Our data likewise showed that both H_2_S donors normalized the inflammatory (p-IKBα), neurotrophic (BDNF) variations caused by CCI, increased the expression of CB2R and activated the Nrf2/HO-1 antioxidant pathway in PFC, v-HIP and/or PAG of animals with neuropathic pain. In addition, the blockade of the analgesia produced by high doses of DADS and GYY4137 with AM630 indicated the contribution of the endocannabinoid system in the effects of H_2_S during neuropathic pain, thus supporting the positive interaction between H_2_S and CB2R. Therefore, this study demonstrates the potential use of CB2R agonists combined with H_2_S donors as a possible treatment for peripheral nerve injury-caused neuropathic pain and the associated emotional disturbances.

## 1. Introduction

Neuropathic pain is a chronic disease with clinical features such as allodynia, hyperalgesia, paresthesia and spontaneous pain, among others. Patients with peripheral nerve injury causing neuropathic pain experience high rates of comorbidities, such as depression and anxiety, which can persist for a long time and have a negative impact on patients’ lives [1,2].

Most pharmacological treatments used for the management of peripheral neuropathic pain, including the use of opioids, pregabalin and gabapentin, are ineffective and have serious side effects that limited their therapeutic use [3,4,5]. Therefore, there is a need to identify new therapies for the management of neuropathic pain and its accompanying affective disorders, with few side effects.

The therapeutic potential of the cannabinoid system in different neurological diseases has been widely demonstrated [6]. There are two types of cannabinoid receptors: type 1 (CB1R) and 2 (CB2R) [7,8]. CB1Rs are mostly found in the central nervous system (CNS) [9], and CB2Rs are found in the microglia and immune cells, although they were also detected in the spinal cord and dorsal root ganglia [10]. More recent research further revealed that CB2Rs were also distributed in the hippocampus (HIP), cortex and cerebellum [11].

Interestingly, since CB2R agonists do not cause psychiatric disorders like CB1R agonists, their therapeutic role in pain has been extensively investigated [12,13,14]. Various studies showed that selective agonists of CB2R protected neurons from apoptosis [15] and reduced neuroinflammation after spinal cord injury [16]. Moreover, although some findings reported that CB2R agonists could induce anxiety-like behaviors and that the overexpression of CB2R produced depressive-like behaviors in mice [17,18], the antidepressant and anxiolytic effects of CB2R agonists in animals suffering diabetic neuropathy, inflammatory or osteoarthritis pain have also been proven [12,13,19]. Nevertheless, the possible effects of CB2R agonists in mood disorders associated with the chronic constriction of sciatic nerve (CCI)-provoked neuropathic pain have not been fully investigated.

Previous research demonstrated that the repetitive administration of two hydrogen sulfide (H_2_S) donors, DADS, diallyl disulfide and/or GYY4137, morpholin-4-ium 4-methoxyphenyl (morpholino) phosphinodithioate dichloromethane complex, alleviated neuropathic pain provoked by CCI [20], as well as of neuropathy caused by chemotherapy and osteoarthritic pain besides their accompanying anxiety- and depressive-like behaviors [21,22]. On the other hand, these two H_2_S donors augmented the analgesic effects of opioids and those produced by carbon monoxide (CO) donors or a heme oxygenase 1 (HO-1) inducer in mice with neuropathic and joint pain [23,24]. However, whether treatment with DADS and/or GYY4137 could potentiate the analgesic actions of CB2R agonists, its effects on the emotional disorders and/or regulate the brain levels of CB2R in animals with CCI-induced neuropathic pain remains unknown, being the main objectives of this study.

Oxidative stress and central sensitization are inextricably linked to the development of neuropathic pain and its accompanying mood disorders [25,26]. These associated emotional disorders are also linked with neuroinflammation and high levels of proinflammatory targets such as the nuclear factor κB (NF-κB), of which the activation induces the phosphorylation of kappa-B inhibitor (p-IKBα) in the CNS and PNS [27,28]. Moreover, exogenous melatonin and irisin alleviated neuropathic pain-associated affective disorders and ethanol-induced behavioral deficits by suppressing the NF-κB activation pathway [29,30].

Brain-derived neurotrophic factor (BDNF) is important for controlling the neuroplasticity and neurogenesis in the CNS (cortex, HIP and subventricular zone) [31] and peripheral nervous system (PNS), for example, in the dorsal root ganglia [32]. Thus, previous studies have shown that stress, injury or inflammation could induce the upregulation of BDNF in the PNS [33], and the down- and upregulation in specific brain areas, such as the HIP and periaqueductal gray matter (PAG), respectively [34,35]. Nonetheless, the effects of H_2_S in the brain expression of BDNF of animals with affective-like behaviors linked with chronic pain are not fully known.

Hence, in male mice with anxiodepressive-like behaviors accompanying CCI-incited neuropathic pain, we examined: (1) the result of the combined administration of DADS and GYY4137 with JWH-133 in modulating the nociceptive responses; (2) the reversion of the analgesics properties of JWH-133, DADS and GYY4137 with AM630 (a CB2R antagonist); (3) the anxiolytic and/or antidepressant actions of JWH-133 alone and co-administered with GYY4137; (4) the effects of H_2_S in the expression of p-IKBα, BDNF, CB2R, NRF2 and HO-1 in the PFC, ventral HIP (vHIP) and PAG, areas deeply involved in the control of nociception and emotions [36,37,38,39].

## 2. Materials and Method

### 2.1. Animals

We used male C57BL/6 mice from Envigo Laboratories (Barcelona, Spain) to carry out the experiments. The mice (age, 5–6 weeks; weight, 21–26 g) were kept under standard conditions of light/dark (12/12 h), temperature (22 ± 1 °C), and relative humidity (55 ± 10%), with free access to water and food. The experiments were performed between 9:00 a.m. and 5:00 p.m., after a week of acclimatization and according to the ethical guidelines of the European Commission’s directive (2010/63/EC) and the Spanish Law (RD 53/2013), and were approved by the local Committee of Animal Use and Care of the Autonomous University of Barcelona (ethical code 9863). Maximal efforts to reduce the number and suffering of animals were accomplished.

### 2.2. Neuropathic Pain Induction

CCI was utilized for inducing neuropathic pain. Under isoflurane anesthesia conditions (3% induction, 2.5% maintenance), after the biceps femoris and the gluteus superficialis were separated by blunt dissection, three ligatures (4/0 silk) across the sciatic nerve were performed. The same process, excluding nerve ligation, was employed in the control mice (sham).

### 2.3. Mechanical Allodynia

Mechanical allodynia was assessed by evaluating the hind paw withdrawal reaction to the von Frey filament stimulation, from 0.4 to 3.5 g (North Coast Medical, Inc., San Jose, CA, USA). The mice were positioned in Plexiglas tubes (20 cm high × 9 cm diameter) with a wire grid bottom. Using the up–down paradigm [40], a filament of 0.4 g was applied first, and depending on the animal’s reaction, the strength of the next filament was increased or reduced. The threshold of the response was estimated applying an Excel program (Microsoft Iberia SRL, Barcelona, Spain) that incorporated curve-fitting of the data.

### 2.4. Thermal Hyperalgesia

Thermal hyperalgesia was evaluated by measuring the paw withdrawal latency in response to a radiant heat using the plantar test (Ugo Basile, Varese, Italy) [41]. The mice were arranged in Plexiglas tubes (20 cm high × 9 cm diameter) and positioned on a glass surface. The heat source sited on the plantar surface of the hind paws was activated with a light beam intensity until the paw withdrawal. The paw withdrawal latencies were obtained from the mean of the three separate trials.

### 2.5. Cold Allodynia

Cold allodynia was determined by recording the amount of elevation of each hind paw of the mice exposed to the cold plate (4 ± 0.5 °C) (Ugo Basile, Italy) for 5 min.

In all paradigms, both the ipsilateral and contralateral paws were assessed.

### 2.6. Anxiety-like Behaviors

We used the elevated plus maze (EPM), an X-shaped structure with 2 open and 2 closed arms, to evaluate the anxiety-like behaviors. All arms were 5 cm wide and 35 cm long, and the closed ones had 15 cm high walls. At the beginning of the test, the mouse was placed in the central area of the maze, always looking at the same open arm, and its behavior was recorded with a digital camera for 5 min. Both the number of entries into the open and closed arms were recorded, as well as the percentage of time spent in the open arms.

### 2.7. Depressive-like Behaviors

The tail suspension test (TST) and the forced swimming test (FST) were used to evaluate depressive-like behaviors.

In the TST, an adhesive tape, attached 1 cm from the end of the tail, was used to suspend the animals from a firm structure placed 35 cm from the ground. The animals were recorded for 8 min and their immobility time during the last 6 min was quantified.

In the FST, the mice were placed individually inside a plexiglass tube (25 cm high × 10 cm diameter) containing water at 24 ± 1 °C, up to a height of 10 cm. The mice were recorded for 6 min and their immobility time was measured for the last 4 min.

All these tests were performed by researchers blinded to the experimental conditions and the animals, between 9 and 10 weeks old, were familiarized with the testing room for 1 h before the start of the experiment.

### 2.8. Western Blot

The animals were euthanized by a cervical dislocation at 28 days after surgery (CCI or sham). The brain, obtained by performing a craniotomy, was dissected using a brain matrix. The PFC, HIP and PAG areas were identified following the Paxinos and Franklin’s stereotaxic coordinates [42]. Additionally, vHIP was separated from the dorsal HIP using the methodology described by [43]. The specific areas were extracted by cutting with a clean scalpel or a punch, pushing the metal gently, but firmly, into the tissue. Rocking it back and forth allowed for making the cut and harvesting the region of interest, which was then placed into labeled pre-chilled Eppendorf tubes and stored at –80 °C until further use. The samples from two animals were pooled into one experimental sample to obtain sufficient protein levels to perform Western blot analysis. The expression of p-IKBα, BDNF, CB2R, NRF2 and HO-1 were evaluated. The tissues were sonicated in a cold lysis buffer, the RIPA buffer (Sigma-Aldrich, St. Louis, MO, USA). After 1 h of dissolution (4 °C), the crude homogenate was sonicated (10 s) and centrifuged at 700× *g* (20 min) at 4 °C. After that, 60 µg of the total protein combined with 4x Laemmli loading buffer was loaded onto a 4% stacking/12% separating sodium dodecyl sulfate polyacrylamide gel. The electrophoretic transfer of the proteins onto polyvinylidene fluoride membranes for 120 min was successfully performed. The membranes were blocked with phosphate-buffered saline (PBS; Sigma-Aldrich, MO, USA) containing 5% nonfat dry milk, Tris-buffered saline with Tween 20 containing 5% bovine serum albumin (BSA; Sigma-Aldrich, MO, USA) or 5% nonfat dry milk and PBS with Tween 20 containing 5% BSA (75 min). The membranes were incubated with rabbit primary antibodies anti-p-IKBα (1:150), BDNF (1:150) and NRF2 (1:150) from Abcam (Cambridge, UK); IKBα (1:150) from Cell Signaling Technology (Danvers, MA, USA); HO-1 (1:100) from Enzo Life Sciences (New York, NY, USA); CB2R (1:150) from Cayman Chemical Company (Ann Arbor, MI, USA); and anti-glyceraldehyde-3-phosphate dehydrogenase (GAPDH, 1:5000) from Merck (Billerica, MA, USA) as a loading control, at 4 °C overnight. The blots were incubated at room temperature for 1 h with secondary polyclonal antibodies conjugated to horseradish peroxidase (GE Healthcare, Little Chalfont, Buckinghamshire, UK). The ECL kit (GE, Healthcare, Little Chalfont, Buckinghamshire, UK) was used to detect the proteins, and the Image-J program (National Institutes of Health, Bethesda, MD, USA) for densitometric analysis.

### 2.9. Experiments

Our objective is to find a treatment that inhibits not only the allodynia and hyperalgesia provoked by nerve injury, but also the anxiodepressive-like behaviors accompanying neuropathic pain. Therefore, considering that nerve injury leads to sensorial hypersensitivity from the onset of injury, whereas the associated anxiodepressive-like behaviors are only obvious after several weeks (4–6 weeks) of injury [44], all these experiments were performed at 28 days after CCI.

Firstly, baseline responses were established using the following test sequence: von Frey filaments, plantar and cold plate tests. After baseline measurements, neuropathic pain was induced by CCI, and the animals were tested again at day 28 after surgery using the same sequence as previously.

To examine the impact of H_2_S in the pain reliever actions of JWH-133, a CB2R agonist [6] in neuropathic pain, the antinociceptive effects of the systemic co-administration of small doses of DADS (3.5 mg/kg) or GYY4137 (0.7 mg/kg) with JWH-133, 2 mg/kg, injected intraperitoneally and 5 µg subplantarly, were assessed (*n* = 8 animals/group).

The impact of the intraperitoneal co-injection of 0.7 mg/kg GYY4137 with 2 mg/kg JWH-133 in the anxiodepressive-like behaviors accompanying neuropathic pain were also determined in parallel within different groups of animals (*n* = 8 animals per group). These experiments were performed at day 28 after CCI induction. The doses of JWH-133 were chosen from the dose-response curves of this study and those of the DADS and GYY4137 from preceding works [23]. In all cases, the animals were treated with DADS and GYY4137 15 min prior to JWH-133 injection, and the tests were accomplished after 45 min.

The reversal of the painkilling effects produced by 50 mg/kg or 150 µg JWH-133, 30 mg/kg DADS or 24 mg/kg GYY4137 with 3 mg/kg or 90 µg AM630, administered intraperitoneally or subplantarly, were also tested. The JWH-133 and H_2_S donors were administered 15 min prior to AM630, and the tests were performed 45 min afterward (*n* = 8 mice/group). The doses of DADS, GYY4137 and AM630 were chosen in accordance with previous findings [23].

Lastly, using the samples of animals that have undergone functional tests, the effects of DADS or GYY4137 on the protein levels of p-IKBα, BDNF, CB2R, NRF2 and HO-1 in the PFC, v-HIP and PAG were evaluated. The controls were sham-operated mice administered with vehicle. In these experiments, 4 samples/group were evaluated based on the sample size analysis performed with the data obtained in a pilot experiment, accepting a risk of α = 0.05 and β = 0.2 in a two-tailed test.

### 2.10. Drugs

Both GYY4137 and DADS from Sigma-Aldrich (St. Louis, MO, USA) were dissolved in 0.9% saline, and given intraperitoneally (10 mL/kg) 1 h before testing [21]. JWH-133 (Sigma-Aldrich, St. Louis, MO, USA) dissolved in a saline solution with 1% Tween 80 (Sigma-Aldrich, St. Louis, MO, USA), and AM630 (LabClinics (Barcelona, Spain) dissolved in a mixed solution containing 90% saline, 5% DMSO and 5% Tween 80, were both injected in a volume of 10 mL/kg (intraperitoneal) or 30 µL (subplantar) [45,46].

All drugs were freshly prepared, and for each group treated with a drug, the respective control group received the same volume of the corresponding vehicle.

### 2.11. Statistical Analyses

We used the Prism 8.0 (Graphpad, La Jolla, CA, USA) and the SPSS (version 28, IBM, Madrid, Spain) programs for the statistical analysis. The normal distribution of the data was determined using the Kolmogorov–Smirnov test, and the sample size was calculated using GRANMO program. A two-way analysis of variance (ANOVA) was used to evaluate the antinociceptive actions of JWH-133 and the anxiolytic and antidepressant effects of JWH-133 alone and with GYY4137. A one-way ANOVA followed by a Tukey test was employed to see the effects of DADS and GYY4137 alone or with JWH-133 or AM630, as well as the impact of H_2_S donors on protein levels. The results are presented as the mean values ± standard error of the mean (SEM). A *p* value < 0.05 was considered significant.

## 3. Results

### 3.1. The Antinociceptive Effects of JWH-133 during Neuropathic Pain

**Figure 1:**
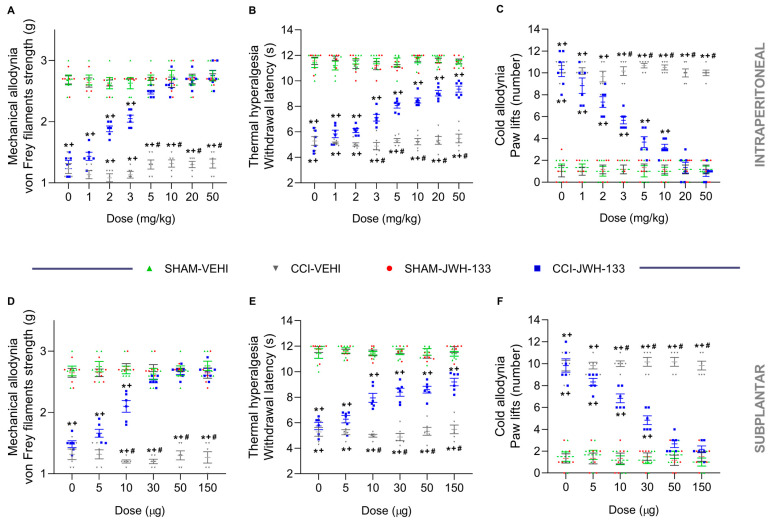
Effects of the intraperitoneal and subplantar administration of JWH-133 on the allodynia and hyperalgesia generated by CCI. Effects of several doses of JWH-133 intraperitoneally (**A**–**C**) and subplantarly (**D**–**F**) administered on the mechanical allodynia (**A**,**D**), thermal hyperalgesia (**B**,**E**) and cold allodynia (**C**,**F**) caused by CCI in the ipsilateral paws. The effects of JWH-133 or vehicle (VEHI) in the ipsilateral paws of sham-operated animals and those of the vehicle in the ipsilateral paws of CCI mice were also represented. For each assay, the symbols denote significant changes versus, * SHAM–VEHI, + SHAM–JWH-133 and # CCI–JWH-133 (*p* < 0.05; one-way ANOVA followed by Tukey test). Results are presented as the mean values ± SEM; *n* = 8 animals/group.

Our results showed that JWH-133, given intraperitoneally (1–50 mg/kg) and subplantarly (5–150 µg) dose-dependently inhibited the allodynia and hyperalgesia generated by CCI (Figure 1).

For all tests, the two-way ANOVA demonstrated the significant effects of the dose and group, and their interaction (*p* < 0.001).

Nerve injury reduced the threshold of paw withdrawal to a mechanical stimulus in comparison to sham-operated mice treated with the vehicle (*p* < 0.001, one-way ANOVA) (Figure 1A,D). Mechanical allodynia was progressively reduced with the administration of 1, 2 and 3 mg/kg or 5 and 10 µg of JWH-133, and was totally inhibited with 5, 10, 20 and 50 mg/kg or 30, 50 and 150 µg of this drug, given intraperitoneally or subplantarly (*p* < 0.001; one-way ANOVA vs. CCI vehicle-treated mice).

Nerve injury also significantly decreased the threshold for evoking paw withdrawal to a thermal stimulus (*p* < 0.001; one-way ANOVA) (Figure 1B,E). Thermal hyperalgesia was gradually reduced with the administration of 1 to 50 mg/kg or 5 to 150 µg of JWH-133 (*p* < 0.001; one-way ANOVA vs. CCI vehicle-treated mice).

The increased paw lift number provoked by the cold thermal stimulation was detected in CCI mice administered with the vehicle (*p* < 0.001; one-way ANOVA vs. sham-operated mice) (Figure 1C,F). Thermal allodynia was also gradually decreased with the administration of 1, 2, 3, 5 and 10 mg/kg or 5, 10 and 30 µg of JWH-133 and was entirely inhibited with 20 and 50 mg/kg or 50 and 150 µg of this drug injected intraperitoneally or subplantarly (*p* < 0.001; one-way ANOVA vs. CCI vehicle-treated mice).

JWH-133 or vehicle had no effect on the ipsilateral paws of sham-operated mice (Figure 1), nor the contralateral paws of sham-operated or CCI mice.

### 3.2. The Analgesic Effects of JWH-133 in Animals with Neuropathic Pain Co-Treated with DADS or GYY4137

**Figure 2:**
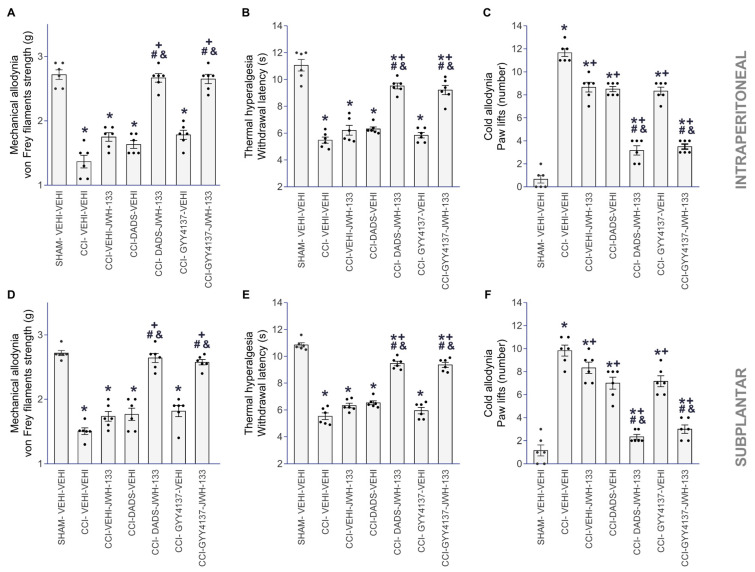
The analgesic actions of JWH-133 co-administered with DADS or GYY4137 in CCI mice. Effects of the co-administration of 3.5 mg/kg of DADS or 0.7 mg/kg of GYY4137 with 2 mg/kg or 5 µg of JWH-133, injected intraperitoneally or subplantarly, on the mechanical allodynia (**A**,**D**), thermal hyperalgesia (**B**,**E**) and cold allodynia (**C**,**F**) caused by CCI in the ipsilateral paws are displayed. The actions of these drugs injected alone are also presented. For each assay, the symbols denote significant changes versus * SHAM–VEHI–VEHI, + CCI–VEHI–VEHI, # CCI–VEHI–JWH-133 and & CCI–DADS–VEHI or CCI–GYY4137–VEHI (*p <* 0.05; a one-way ANOVA, followed by Tukey test). VEHI (vehicle). The results are presented as the mean values ± SEM; *n* = 8 animals/group.

The antiallodynic and antihyperalgesic activities produced by the systemic injection of 3.5 mg/kg or 0.7 mg/kg of DADS or GYY4137 mixed with JWH-133, intraperitoneally (2 mg/kg) or subplantarly (5 µg), were evaluated (Figure 2).

The administration of DADS or GYY4137 significantly improved the mechanical (Figure 2A,D) and cold antiallodynic effects (Figure 2C,F), in addition to the thermal antihyperalgesic effects (Figure 2B,E) made by JWH-133, systemically or locally injected, as related with the effects produced by the vehicle, JWH-133, DADS or GYY4137 given alone (one-way ANOVA, *p* < 0.001).

DADS or GYY4137, administered separately and combined with JWH-133, intraperitoneally or subplantarly, had no impact on the contralateral paws of CCI- or sham-operated mice, nor on the ipsilateral paws of sham-operated animals.

### 3.3. Reversion of the Antinociceptive Actions of JWH-133, DADS and GYY4137 with AM630

**Figure 3:**
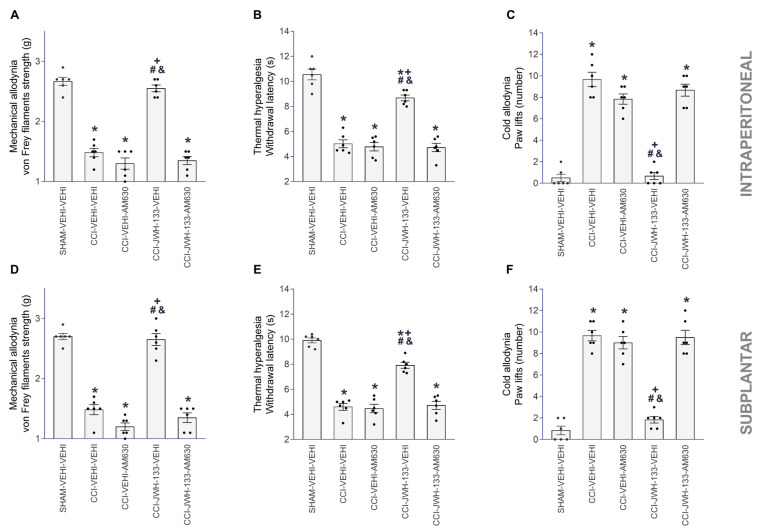
Reversal of the antiallodynic and antihyperalgesic effects of JWH-133 with AM630 in CCI mice. Effects of AM630 injected intraperitoneally (3 mg/kg) or subplantarly (90 µg) on the inhibition of the mechanical allodynia (**A**,**D**), thermal hyperalgesia (**B**,**E**) and cold allodynia (**C**,**F**) produced by the intraperitoneal (50 mg/kg) or subplantar (150 µg) administration of JWH-133 in the ipsilateral paws of CCI mice. For each assay and administration route, the symbols denote significant changes versus * SHAM–VEHI–VEHI, + CCI–VEHI–VEHI, # CCI–VEHI–AM630 and & CCI–JWH-133–AM630 (*p* < 0.05; one-way ANOVA, followed by Tukey test). VEHI (vehicle). The results are presented as the mean values ± SEM; *n* = 8 animals/treatment.

The intraperitoneal (3 mg/kg) and subplantar (90 µg) administration of the CB2R antagonist AM630 reversed the mechanical (Figure 3A,D) and cold antiallodynic actions (Figure 3C,F), as well as thermal antihyperalgesic activities (Figure 3B,E) produced by JWH-133 injected intraperitoneally (50 mg/kg) or subplantarly (150 µg), respectively (one-way ANOVA, *p* < 0.001).

**Figure 4:**
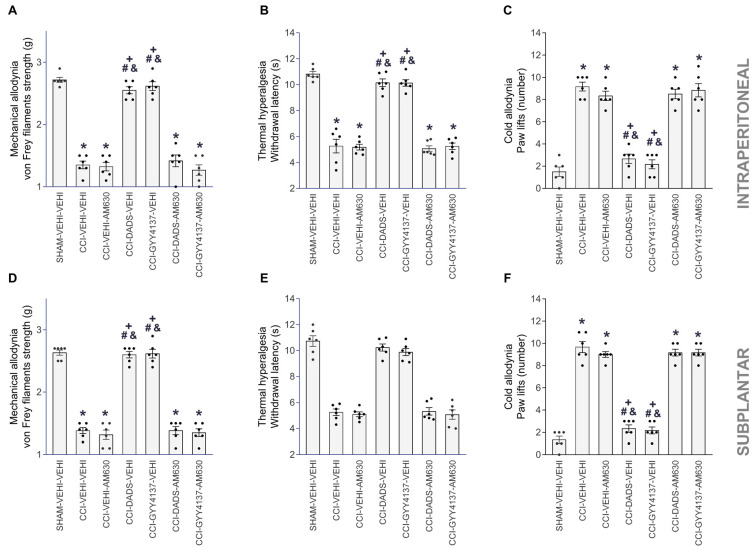
AM630 reversed the antiallodynic and antihyperalgesic effects of DADS and GYY4137 during neuropathic pain. The intraperitoneal (3 mg/kg) and subplantar (90 µg) administration of AM630 reversed the inhibition induced by 30 mg/kg of DADS or 24 mg/kg of GYY4137 in the mechanical allodynia (**A**,**D**), thermal hyperalgesia (**B**,**E**) and cold allodynia (**C**,**F**), provoked by CCI. For each assay and administration route, the symbols denote significant changes versus * SHAM–VEHI–VEHI, + CCI–VEHI–VEHI, # CCI–VEHI–AM630 and & CCI–DADS–AM630 or CCI–GYY4137–AM630 (*p* < 0.05; one-way ANOVA, followed by Tukey test). VEHI (vehicle). The results are presented as the mean values ± SEM; *n* = 8 animals/treatment.

To examine the feasible contributions of the endocannabinoid system in the painkilling properties of H_2_S donors, we assessed if AM630 could also reverse the painkiller effects produced by high doses of DADS or GYY4137 in animals experiencing neuropathic pain. Our results showed that the mechanical antiallodynic (Figure 4A,D), thermal antihyperalgesic (Figure 4B,E), and cold antiallodynic effects (Figure 4C,F) of 30 mg/kg DADS and 24 mg/kg GYY4137 were blocked with 3 mg/kg (intraperitoneal) or 90 µg (subplantar) of AM630 (one-way ANOVA, *p* < 0.001).

Furthermore, the treatment with VEHI, DADS, GYY4137, JWH-133 or AM630, alone or combined, given intraperitoneally or subplantarly, did not have any impact on the contralateral paws of the CCI animals nor in the contralateral and ipsilateral paws of sham-operated mice.

### 3.4. Effects of JWH-133 Alone or Combined with GYY4137 on the Neuropathic-Pain-Associated Anxiodepressive-liked Behaviors

Considering the high antinociceptive effectiveness of the combined systemic treatment of GYY4137 and JWH-133, we assessed the possible anxiolytic and/or antidepressant actions produced by this combination in animals with neuropathic pain.

**Figure 5:**
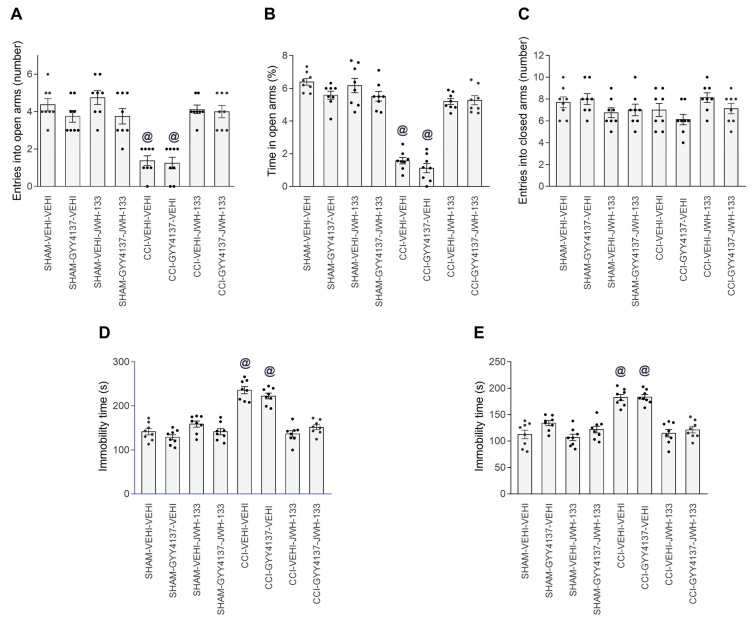
Combined treatment of GYY4137 with JWH-133 inhibited the anxiety- and depressive-like behaviors linked with neuropathic pain. Effects of the intraperitoneal administration of GYY4137 (0.7 mg/kg) and JWH-133 (2 mg/kg), alone and combined, on the anxiety- and depressive-like behaviors associated with CCI-provoked neuropathic pain. The effects of GYY4137, JWH-133, GYY4137 plus JWH-133 or the vehicle (VEHI) in sham-operated mice are also displayed. In the EPM test, the number of entrances into the open arms (**A**), proportion of time spent in the open arms (**B**) and the quantity of entrances into the closed arms (**C**) are shown. In the TST (**D**) and FST (**E**), the immobility times (s) are displayed. For each assay, @ denotes the significant differences versus the rest of the groups (*p* < 0.05; one-way ANOVA, followed by Tukey test). The results are presented as the mean values ± SEM; *n* = 8 animals/group.

In the EPM test, the two-way ANOVA showed significant effects of surgery and treatment, as well as their interaction (*p* < 0.001) regarding the number of entries and the time that the animals remained in the open arms, but not in the number of times that they entered the closed arms. Therefore, the results showed that 2 mg/kg of JWH-133 alone and combined with 0.7 mg/kg of GYY4137 both regularized the reduced quantity of entrances into the open arms (one-way ANOVA, *p* < 0.001; Figure 5A) and the short time spent in them by the CCI animals treated with vehicle (one-way ANOVA, *p* < 0.0031; Figure 5B). The single treatment with GYY4137 did not change the number of entries and the time spent in the open arms of CCI mice. No differences in the number of closed arm entries were identified between the groups (Figure 5C).

The two-way ANOVA also demonstrated significant effects of surgery, treatment, and their interaction (*p* < 0.001) in the TST and FST. Therefore, the elevated immobility time of CCI mice treated with the vehicle in the TST (*p* < 0.001; one-way ANOVA; Figure 5D) and FST (*p* < 0.001; one-way ANOVA; Figure 5E) was decreased by JWH-133 alone or in combination with GYY4137. In these tests, the non-effects of GYY4137 alone were observed in CCI mice.

In addition, the non-effects of treatment with GYY4137, JWH-133 or GYY4137 plus JWH-133 were observed in the EPM, TST or FST of sham-operated mice (Figure 5).

These results revealed the anxiolytic and antidepressant actions produced by a CB2R agonist administered alone and combined with a H_2_S donor in animals with affective disorders associated with neuropathic pain.

### 3.5. Effects of DADS and GYY4137 on the p-IKBα, BDNF, CB2R, NRF2 and HO-1 Levels in the PFC, vHIP and PAG of Mice with Neuropathic Pain

**Figure 6:**
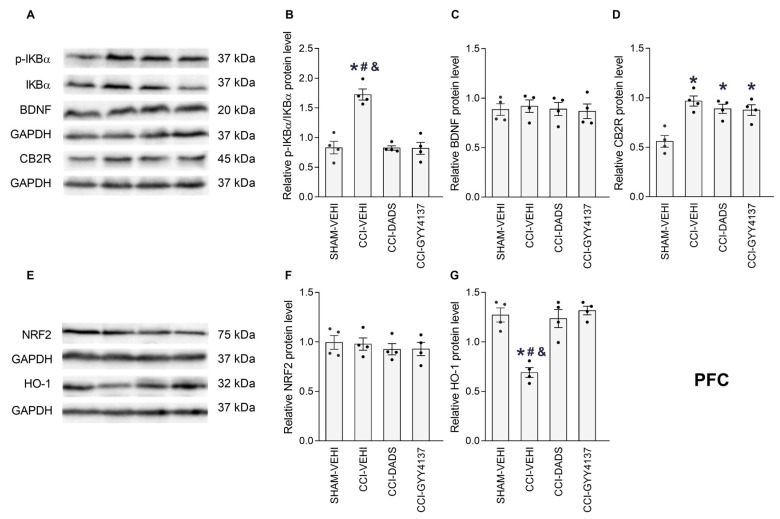
The effects of treatment with DADS and GYY4137 on the expression of p-IKBα, BDNF, CB2R, NRF2 and HO-1 in the PFC of mice with neuropathic pain. Both treatments reversed the up-regulation of p-IKBα (**B**) and the downregulation of HO-1 (**G**), and further maintained the high levels of CB2R (**D**) in CCI mice. No variations in the BDNF (**C**) or NRF2 expressions (**F**) were identified. Sham-operated mice treated with the vehicle (VEHI) were employed as the control group. All proteins are expressed relative to the GAPDH protein levels, except p-IKBα, which is relative to IKBα. Representative blots for p-IKBα, BDNF and CB2R (**A**), and for NRF2 and HO-1 (**E**) are shown. In all pictures, the symbols denote significant changes versus, * sham-operated mice treated with VEHI, # CCI mice treated whit DADS and & CCI mice treated GYY4137 (*p* < 0.05; one-way ANOVA, followed by Tukey test). The results are presented as the mean values ± SEM; *n* = 4 samples/group.

**Figure 7:**
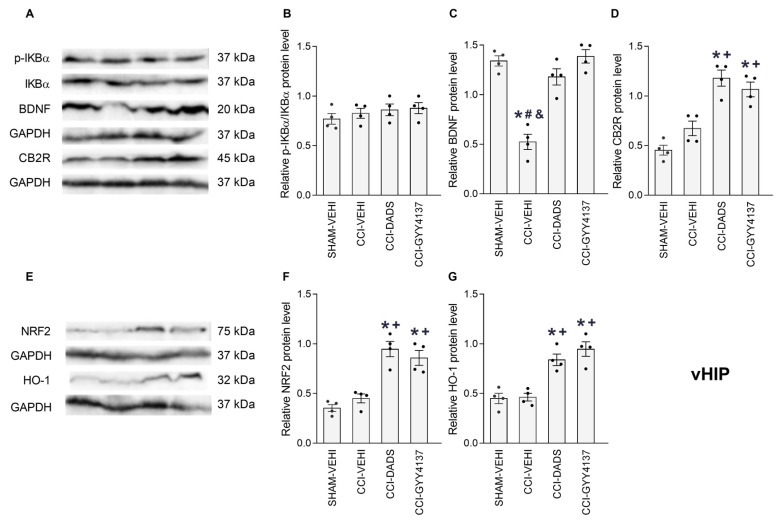
The effects of treatment with DADS and GYY4137 on the expression of p-IKBα, BDNF, CB2R, NRF2 and HO-1 in the vHIP of mice with neuropathic pain. Both treatments reversed the downregulation of BDNF (**C**), and increased the expression of CB2R (**D**), NRF2 (**F**) and HO-1 (**G**) in CCI mice. No variations in the p-IKBα expression (**B**) were identified. Sham-operated mice treated with the vehicle (VEHI) were employed as the control group. All proteins are expressed relative to the GAPDH protein levels, except p-IKBα, which is relative to IKBα. Representative blots for p-IKBα, BDNF and CB2R (**A**), and for NRF2 and HO-1 (**E**) are shown. In all pictures, the symbols denote significant changes versus, * sham-operated mice treated with VEHI, + CCI mice treated with VEHI, # CCI mice treated DADS and & CCI mice treated GYY4137 (*p* < 0.05; one-way ANOVA, followed by Tukey test). The results are presented as the mean values ± SEM; *n* = 4 samples/group.

**Figure 8:**
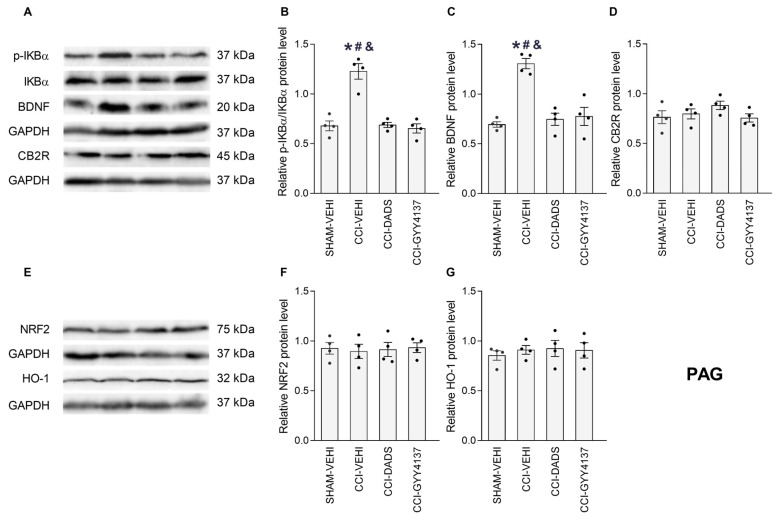
The effects of treatment with DADS and GYY4137 on the expression of p-IKBα, BDNF, CB2R, NRF2 and HO-1 in the PAG of mice with neuropathic pain. Both treatments reversed the upregulation of p-IKBα (**B**) and BDNF (**C**) induced by CCI. No variations in the CB2R (**D**), NRF2 (**F**) and HO-1 (**G**) were identified. Sham-operated mice treated with the vehicle (VEHI) were employed as the control group. All proteins are expressed relative to the GAPDH protein levels, except p-IKBα, which is relative to IKBα. Representative blots for p-IKBα, BDNF and CB2R (**A**), and for NRF2 and HO-1 (**E**) are shown. In all pictures, the symbols denote significant changes versus, * sham-operated mice treated with VEH, # CCI mice treated whit DADS and & CCI mice treated GYY4137 (*p* < 0.05; one-way ANOVA, followed by Tukey test). The results are presented as the mean values ± SEM; *n* = 4 samples/group.

We analyzed the impact of DADS and GYY4137 treatments on the expression of p-IKBα, BDNF, CB2R, NRF2 and HO-1 in the PFC (Figure 6), vHIP (Figure 7) and PAG (Figure 8) of CCI animals. Sciatic nerve injury enhanced the expression of p-IKBα in PFC (Figure 6B; *p* < 0.001; one-way ANOVA) and PAG (Figure 8B; *p* < 0.001; one-way ANOVA) and DADS, as well as GYY4137, reversed these effects. Both treatments further normalized the low and high expression of BDNF in the vHIP (Figure 7C; *p* < 0.007, one-way ANOVA) and PAG (Figure 8C; *p* < 0.001, one-way ANOVA). Moreover, DADS and GYY4137 retained elevated CB2R levels in the PFC of CCI mice (Figure 6D; *p* < 0.007, one-way ANOVA) and activated its expression in the vHIP of these animals (Figure 7D; *p* < 0.001, one-way ANOVA). Both H_2_S donors increased the NRF2 expression in the vHIP (Figure 7F; *p* < 0.001, one-way ANOVA), avoided the downregulation of HO-1 in PFC (Figure 6G; *p* < 0.001, one-way ANOVA) and increased its expression in the vHIP (Figure 7G; *p* < 0.001, one-way ANOVA). No changes in the expression of p-IKBα in the vHIP (Figure 7B), BDNF in the PFC (Figure 6C), CB2R in the PAG (Figure 8D), NRF2 in the PFC (Figure 6F) or PAG (Figure 8F), nor HO-1 in the PAG (Figure 8G) were identified.

## 4. Discussion

Our results demonstrated that pretreatment with H_2_S donors significantly improved the antinociceptive effects of JWH-133, a CB2R agonist, and preserved its anxiolytic and antidepressant actions in mice with mood disorders associated with neuropathic pain. The analgesic actions of JWH-133, DADS and GYY4137 were reversed by the local and systemic administration of the CB2R antagonist, AM630. Moreover, both H_2_S donors normalized the inflammatory, neurotrophic and oxidative replies elicited by CCI, as well as modulated the expression of CB2R in the PFC, vHIP and/or PAG of animals with neuropathic pain.

Our results showed that JWH-133, given intraperitoneally or subplantarly, dose-dependently diminished the sciatic nerve injury-incited allodynia and hyperalgesia. In accordance, previous studies using JWH-133 and/or other CB2R agonists also demonstrated that their systemic and/or local administration decreased the allodynia and hyperalgesia in different models of neuropathic pain caused by nerve injury, brachial plexus avulsion or cisplatin injection [47,48,49]. Our data further revealed that the intraperitoneal and subplantar injection of AM630 abolished the analgesic actions of JWH-133, validating the specificity of the central and peripheral pain-relieving actions of this CB2R agonist under neuropathic pain conditions. Studies performed with CB2R-knockout mice supported these findings by demonstrating that the allodynia and hyperalgesia produced by nerve injury increased in these animals [50,51].

Different studies revealed a potentiation of the pain-relieving activity of CB2R agonists through the co-administration of Nrf2 transcription factor activators or HO-1 enzyme inducers in animals with inflammatory pain and neuropathy associated with diabetes [47]. Nevertheless, the effects of H_2_S donors in the antinociceptive effects of CB2R, as well as in their plausible anxiolytic and/or antidepressant actions in animals with emotional disturbances accompanying neuropathic pain, have not been previously investigated. For the first time, this study showed that DADS and GYY4137, two slowly H_2_S-releasing compounds, augmented the antinociceptive actions of JWH-133, administered systemically or locally, and further preserved the anxiolytic and antidepressant actions performed by this CB2R agonist in CCI mice. On the contrary, the co-administration of CB2R agonists with an HO-1-inducer compound decreased the pain-killing actions of CB2R in CCI mice [47]. Thus, reporting the different effects produced by the two gaseous neurotransmitters, H_2_S and CO, in modulating the actions of CB2R agonists under sciatic nerve injury-induced neuropathic pain and highlighting the co-treatment of H_2_S with CB2R as a good strategy for relieving CCI-generated neuropathic pain. Interestingly, the analgesia induced by high doses of DADS and GYY4137 administered systemically was reversed by AM630, given intraperitoneally and subplantarly, indicating that central and peripheral endocannabinoids might be involved in the antinociceptive actions of H_2_S donors, as it occurs with the opioid system [23].

The endogenous cannabinoid system is also critical in the regulation of affective diseases [18]. Moreover, CB2Rs are found in several brain areas participating in the control of emotional behaviors, such as the amygdala, PFC and HIP, thus sustaining the potential use of CB2Rs in modulating affective disorders [6,18,52]. In this way, our data showed that the intraperitoneal administration of 2 mg/kg JWH-133 did not produce any effect on the EPM, TST or FST of sham-operated mice, but it was capable of reversing the reduced number of entrances and time spent in the EPM open arms, and also reduced the immobility time of CCI mice in the TST and FST. Thus, this showed the anxiolytic and antidepressant properties of this CB2R agonist under neuropathic pain conditions. In accordance with our results, JWH-133 injected into CB2R-gene-knockout mice did not reverse the anxiogenic-like behaviors associated with neuropathic pain caused by the partial sciatic nerve ligation [14], and the administration of another CB2R agonist (GW405833) also reduced the depressive-like behaviors in rats with mononeuropathy [52].

In addition, this study revealed that the acute treatment with a low dose of GYY4137 did not impede the emotional disorders associated with neuropathic pain, the anxiety- nor depressive-liked behaviors, confirming that the repetitive administration of slow H_2_S-releasing donors are needed to modulate the affective deficits under chronic pain conditions [21]. Interestingly, the combining treatment of GYY4137 with JWH-133 maintained the positive emotive actions of the CB2R agonist in CCI mice, suggesting that the effects produced by this combined therapy on mood disorders were mainly produced by JWH-133, while the enhanced analgesic actions of GYY4137 combined with JWH-133 in CCI mice seems to be the result of a positive interaction between both systems, H_2_S and CB2R.

In this research, we furthermore analyzed the impact of DADS and GYY4137 on the p-IKBα, BDNF, CB2R, NRF2 and HO-1 protein levels in the PFC, vHIP and PAG of animals with nerve injury-provoked neuropathy. Many studies indicate that the induction and maintenance of neuropathic pain is linked to an inflammatory element [53,54]. Accordingly, elevated levels of p-IKBα were detected in the PFC and PAG of CCI mice that were inhibited by DADS and GYY4137, revealing the anti-inflammatory activity of these treatments, which can contribute to enhance the analgesic effects of CB2R agonists. It has been demonstrated that the excessive synthesis of inflammatory mediators induced by CCI produces a depletion of endogenous cannabinoids in the PFC and abnormal changes of endocannabinoid signaling during neuropathic pain [55]. Therefore, considering that the administration of ATB-352, a H_2_S donor, inhibits the enzymes involved in the degradation of the endogenous cannabinoids in the gut [56], we speculated that the reduction of the inflammatory responses produced by DADS and GYY4137 in the PFC and PAG of CCI mice might inhibit the enzyme monoacylglycerol lipase (MGL), involved in the degradation of 2-arachidonoyl glycerol (2-AG), resulting in the normalization of the low levels of this endocannabinoid in the brain, and thus explaining the enhancement of the analgesic effects of JWH-133 produced by DADS and GYY4137 during neuropathic pain. In accordance, it has been demonstrated that the inhibition of MGL with JZL184 increased the 2-AG levels in the brain and reduced neuropathic pain induced by CCI [57,58]. Nonetheless, additional experiments are needed to confirm this theory.

BDNF is a neurotrophic factor with multiple roles in the body, including the regulation of neuronal survival, shaping neurons and synaptic plasticity [59]. It is also involved in the pathophysiology of pain, depression and anxiety [60,61]. BDNF is initially produced as a precursor (pro-BDNF), which is then broken down to produce mature BDNF (mBDNF). ProBDNF and mBDNF have different functions, while mBDNF promotes neuronal survival, differentiation and synaptic plasticity, pro-BDNF induces apoptosis and dendritic spine plasticity [62]. In this study, we evaluated the effects of DADS and GYY4137 on the expression of mBDNF in the PFC, vHIP and PAG of animals with neuropathic pain. Our results demonstrated that CCI increased the mBDNF expression in the PAG and decreased them in the vHIP. In agreement with this, Guo et al., 2006 [63], also demonstrated the high levels of mBDNF in the PAG and rostral ventromedial medulla of animals with peripheral injury, and demonstrated that the hypersensitization induced by central mBDNF is mediated by the activation of the TrkB-NMDAR-dependent excitatory transmission [64]. In this study, we also demonstrated that DADS and GYY4137 normalized the high expression of mBDNF in the PAG, suggesting that these actions might contribute to the modulation of nociception produced by these H_2_S donors during neuropathic pain. Other authors indicated that peripheral proBDNF, but not mBDNF, contributes to pain hypersensitivity induced by peripheral inflammation [65], thus revealing the different effects induced by peripheral and/or central pro- and mBDNF in regulating pain.

It is also established that BDNF plays an important role in regulating anxiety and depression. Several studies reveal that pro- and mBDNF might develop different functions in controlling these emotional behaviors [66]. Indeed, the upregulation of proBDNF in the hippocampus of animals with anxiety- and depressive-like behaviors associated with peripheral inflammation [67], and the downregulation of mBDNF in the anterior cingulate cortex of mice with anxiodepressive-like behaviors accompanying neuropathic pain [68], support the different roles played by both forms of BDNF in regulating these affective disorders. Our results likewise demonstrated the downregulation of mBDNF in the vHIP of animals with anxiety- and depressive-like behaviors linked with CCI-induced neuropathic pain. Moreover, both H_2_S donors regularized the low levels of BDNF in the v-HIP, contributing to the modulation of the affective disorders produced by them during neuropathic pain [69]. A limitation of this study is the non-evaluation of the proBDNF levels in the PFC, vHIP and PAG, which would have allowed us to know its role in the effects of DADS and GYY4137.

In agreement with another study that has shown increased spinal levels of CB2R during neuropathic pain [70], the high levels of this receptor were displayed in the PFC of CCI mice. Interestingly, both DADS and GYY4137 treatments preserved the upregulation of CB2R in PFC, and augmented its expression in the vHIP of CCI mice, thus explaining the improved painkiller actions and the conservation of the anxiolytic and/or the antidepressant actions of JWH-133 in CCI animals co-treated with GYY4137.

Oxidative stress is also important in the development and preservation of chronic pain [25,71]. In fact, the high levels of oxidative stress markers were observed in the PNS and CNS of animals with neuropathic pain [72,73]. In agreement with these data, low levels of HO-1 were detected in the PFC of CCI mice, and both H_2_S donors normalized this downregulation and enhanced the expression of NRF2 and HO-1 in the vHIP. These findings agreed with the upregulation of HO-1 stimulated by GYY4137 in the medial septum of nerve-injured animals [23]. Our results highlight the antioxidant actions of slow H_2_S releasers in the CNS of mice with neuropathic pain, suggesting that these properties might also contribute to potentiate the analgesic actions of CB2R agonists during neuropathic pain.

It is well recognized that nerve-injury-induced neuropathic pain altered the gastrointestinal microbiota [74,75,76] and that the gut microbiota influences neuropathic pain through the modulation of proinflammatory and anti-inflammatory T cells [77]. Moreover, alterations in the composition of the intestinal microbiota have been characterized in depressed patients [78], and studies on animals demonstrated that gut microbiota dysbiosis is associated with depressive- and anxiety-like behaviors [79,80]. In addition, probiotic intervention has been shown to reduce the anxiodepressive-like behaviors in stressed rats [81] and improve mood in depressive patients [82]. Therefore, and considering that the dysfunctions of intestinal microbiota led to reductions in endogenous H_2_S [83] and the paucity of the precursors of the endocannabinoid system [84], while the administration of H_2_S restored the intestinal microbiota biofilm and stabilized the microbiome–mucosa interface [85]. We postulated that the possible reversion of gut dysbiosis produced by DADS and GYY4137 treatments might normalize the endogenous H_2_S levels and/or the endocannabinoid precursors, thus supporting the enhanced analgesic effects of JWH-133 and the preservation of its anxiolytic and antidepressant properties in CCI animals co-treated with DADS or GYY4137. Nevertheless, further experiments are needed to demonstrate this hypothesis.

## 5. Conclusions

This study demonstrated an enhancement in the antinociceptive effects of JWH-133 induced by H_2_S donors, and revealed the anxiolytic and antidepressant effects of JWH-133 combined with GYY4137 in animals with mood disorders associated with neuropathic pain. The mechanism by which H_2_S potentiates the analgesic effects of CB2R agonists might be related to the preservation of the high levels of CB2R, together with the inhibition of inflammation, oxidative stress and the BDNF overexpression produced by DADS and GYY4137 in the PFC and/or PAG of animals with neuropathic pain. This study also revealed the participation of the endogenous cannabinoid system in the DADS and GYY4137 pain-reliever effects. Finally, based on our findings and considering the lack of tolerance or addiction liability of CB2R agonists [6] and the beneficial effects of H_2_S on the gastrointestinal system [85], we propose the co-treatment of CB2R agonists plus H_2_S donors as a potential effective therapy for peripheral nerve injury-caused neuropathic pain and the associated emotional disturbances, with few side effects.

## Data Availability

Data is contained within the article.
